# Improved endoglucanase production and mycelial biomass of some ericoid fungi

**DOI:** 10.1186/s13568-016-0312-y

**Published:** 2017-01-03

**Authors:** O. R. Adeoyo, B. I. Pletschke, J. F. Dames

**Affiliations:** Department of Biochemistry and Microbiology, Rhodes University, P.O. Box 94, Grahamstown, 6140 South Africa

**Keywords:** Endoglucanase, Ericoid, Endophytic fungi, Mycelial biomass

## Abstract

Fungal species associated with ericaceous plant roots produce a number of enzymes and other bio-active metabolites in order to enhance survival of their host plants in natural environments. This study focussed on endoglucanase production from root associated ericoid mycorrhizal and dark septate endophytic fungal isolates. Out of the five fungal isolates screened, *Leohumicola* sp. (ChemRU330/PPRI 13195) had the highest relative enzyme activity and was tested along with isolates belonging to Hyloscyphaceae (EdRU083/PPRI 17284) and Leotiomycetes (EdRU002/PPRI 17261) for endoglucanase production under different pH and nutritional conditions that included: carbon sources, nitrogen sources and metal ions, at an optimum temperature of 28 °C. An optimal of pH 5.0 produced enzyme activity of 3.99, 2.18 and 4.31 (U/mg protein) for isolates EdRU083, EdRU002 and *Leohumicola* sp. respectively. Increased enzyme activities and improved mycelial biomass production were obtained in the presence of supplements such as potassium, sodium, glucose, maltose, cellobiose, tryptone and peptone. While NaFe-EDTA and Co^2+^ inhibited enzyme activity. The potential role of these fungi as a source of novel enzymes is an ongoing objective of this study.

## Introduction

The soils of temperate, boreal forests and heathlands are characteristically enriched with a large number of soil microbes that include ericoid mycorrhizal (ERM) and dark septate root endophytic (DSE) fungi. Roots of ericaceous plants harbour these fungi, conferring eco-physiological benefits to the host (Bizabani and Dames 2015). A mutualistic association is usually formed between the plant family Ericaceae and related fungal members (Cairney and Meharg 2003). Ericoid mycorrhizas are characterized by densely packed intracellular fungal coils that are formed in the epidermal cells of the fine hair roots of their host plant while establishing a loose hyphal network outside of the hair roots (Smith and Read 2008). These thin hyphal coils within the cortical cells serve as interfaces for nutrient exchange between the symbionts (Vohnik et al. 2009). The primary function of the ERM fungus is to facilitate the utilisation of organic complexes as a source of nutrients for their host plant and in return, the fungus acquires photosynthetic carbon from the host for the completion of their life cycle (Pearson and Read 1975 *). Hymenoscyphus ericae* is a major ericoid fungus that has been extensively investigated for its ability to grow on a variety of complex organic substrates that include carboxymethylcellulose (CMC) (Leake and Miles 1996). ECM associations enable host plants to survive in soils characterized by impoverished nutrient status, e.g. acidic, nitrogen and phosphorus deficient soils (Read 1996). The fungi selected for this study were isolated from the host plants belonging to genus *Erica*. The genus *Erica* is a large varied taxon containing approximately 850 species (Oliver 2000). DSE fungi are conidial or sterile ascomycetes that colonize living plant roots without causing apparent negative effects such as tissue disorganization (Jumpponen and Trappe 1998). Several DSE fungi have been found to form symbiotic relationships with their host plant (Usuki and Narisawa 2007). DSE colonization has been identified in about 600 plant species which represent about 320 genera and 114 families (Jumpponen and Trappe 1998).

Enzymes of microbial origin have high biotechnological importance in the processing of food, manufacturing of detergents, pharmaceutical products and in molecular biology (Falch 1991). Extracellular enzymes are produced by ERM and DSE fungi during utilization of soil organic matter facilitating the degradation of organic substrates into simpler units. Under laboratory conditions, these extracellular enzymes are produced in liquid media and can be assayed to quantify the concentration of enzyme produced per millilitre of the filtrate, per minute. In order to assess endoglucanase production in vitro, experimental liquid medium amended with 1% CMC is often used. This mimics the natural pattern of production where hydrolysis of cellulose occurs as a result of the action of cellulolytic microorganisms in soil (Doolotkeldieva and Bobusheva 2011). Culture-based and field studies have confirmed the saprotrophic capabilities of numerous ERM fungi (Cairney and Meharg 2003) and DSE fungi such as *Cadophora*, *Leptodontidium*, *Phialophora* and *Philocephala* (Berthelot et al. 2016). Their effects have been well established in soils, where they secrete enzymes and secondary metabolites that unlock organic complexes to release mineral nutrients for plant uptake. Endoglucanase is a major component of the cellulase complex (Doolotkeldieva and Bobusheva 2011) and can be produced by both ERM and DSE fungi in vitro. Other vital enzymes such as cellulases, amylases, laccases and pectinases may also be produced (Choi et al. 2005), one unit of activity can be defined (for example) as the quantity of enzyme which, for 30 min at 37 °C, catalyses the splitting of 1 mg of glucose under experimental conditions. In this study, the effects of pH and nutritional factors on endoglucanase activity and mycelial biomass production of selected ERM and DSE fungi in liquid culture was investigated.

## Materials and methods

### Culture

Fungal isolates used for this study were cultured from roots of ericaceous plants (*Erica demissa* and *Erica chamissonis*) (Bizabani 2015). *Leohumicola* sp. (Isolate code ChemRU330/Genbank Accession Number KC979127/The South African National Collection of Fungi Accession Number PPRI 17268), and two unidentified fungi belonging to Hyaloscyphaceae (EdRU083/KF225587/PPRI17261) and Leotiomycetes (EdRU002/KF225581/PPRI17284) were obtained from Mycorrhizal Research Laboratory, Rhodes University, Grahamstown. These isolates were maintained on 2% malt extract agar (MEA) for further studies.

### Media and mycelial preparation

Culture media used for culturing and sub-culturing included MEA, which were prepared according to the manufacture’s specification and Modified Melin Norkrans (MMN) Agar with the following composition (g L^−1^): malt extract 3.0; (NH_4_)_2_HPO_4_ 0.25; KH_2_PO_4_ 0.50; MgSO_4_·7H_2_O 0.15; CaCl_2_ 0.05; NaCl 0.025; ZnSO_4_·7H_2_O 0.003; thiamine-HCl 100 µg; and 1.2 mL of FeCl_3_ (1%, w/v) (Marx 1969), autoclaving was conducted at 121 °C for 15 min. Chloramphenicol (0.05 mg/mL) was added to the cooled media (approximately 45 °C) before pouring into plates to prevent bacterial growth. Mycelia MEA plugs (5 mm) were regularly subcultured and incubated at 28 °C for 3 weeks and stored at 4 °C.

### Screening for cellulase producing ericoid fungi

The basal medium (MMN) was amended with CMC, 1.0% (w/v) (Caldwell et al. 2000). The zones of hydrolysis were viewed by flooding the plates with an aqueous solution of Congo Red (1 mg mL^−1^) for 15 min followed by 1 M NaCl for another 15 min after draining the dye. Stabilization was achieved with 1 M HCl (Teather and Wood 1982). The appearance of halo (yellowish zone) around the fungal colony indicated cellulase activity. The strength of activity of each of the isolate was classified based on the diameter of the hydrolytic zone.

### Production of endoglucanase

A 50 ml MMN liquid medium (pH 5.0) supplemented with 0.5% CMC was placed into a 150 mL Erlenmeyer flask; the contents was thoroughly mixed and sterilized at 121 °C for 15 min. The sterilized medium was inoculated with two 5 mm mycelial plugs, and a non-inoculated medium was used as the control. Growth was allowed to proceed at 28 °C for 3 weeks in a rotary incubator shaker at 150 rpm, after which the cultures were filtered using Buchner funnel with Whatman No. 1 filter paper placed on its perforated plate. The filter paper was moistened with sterile milliQ water before used. The crude extract was poured into the cylinder and filtered through the funnel by vacuum suction. Centrifugation was conducted at 10,000×*g* for 15 min to obtain crude enzyme filtrates.

### Assay for extracellular endoglucanase

A dinitrosalicylic acid (DNS) assay was conducted using the method described by Miller (1959). A CMC (1%, w/v) was each added to a volume of 10 mL citrate–phosphate buffer (pH 5.0) in a Schott bottle and stirred until completely dissolved. A volume of 100 µL of crude enzyme and uninoculated control was added to separate 300 µL of CMC containing medium in triplicate while blank sample contained 400 µL buffer. All samples were incubated at 37 °C for 30 min followed by centrifugation at 6000×*g* 2 min. A 300 µL of DNS was added to 150 µL of each supernatant sample. This was later followed by boiling on a heating block at 100 °C for 5 min after which it was cooled on ice for 5 min. A volume of 250 µL of each sample and control was distributed into a 96-well plate and read with the aid of a spectrophotometer (Synergy Mx) at a wavelength of 540 nm. A unit of endoglucanase activity was defined as the amount of enzyme that liberated 1 µmol of glucose per minute under standard assay condition using glucose standard curve.

### Effect of media manipulation on endoglucanase and mycelial biomass production

The effect of pH on the production of endoglucanase was carried out by adjusting the pH to 3.0, 4.0, 5.0, 6.0, 7.0 and 8.0 using HCl or NaOH (The incubation was done at 28 °C for 3 weeks, and enzyme activity was determined as described previously). Effect of carbon and nitrogen sources on the production of endoglucanase was conducted using either (1.0%, w/v) glucose, maltose, sucrose, cellobiose, starch as supplements and either (1.0%, w/v) peptone, yeast extract, malt extract or tryptone as supplements. The production media were also supplemented with either (0.1%, w/v) Na^+^, K^+^, Zn^2+^, Mn^2+^, and Co^2+^ supplied in sulphate form while 0.1% FeNa-EDTA (ferric EDTA) was supplied as ethylenediamine-tetraacetic acid iron(III) sodium salt to determine the effect of metal ions. Mycelial biomass obtained from each of the treatments was expressed as dry weight after drying in a hot air oven at 80 °C for 16 h (Sunitha et al. 2012). Protein content was measured according to the method described by Bradford (1976) using Bovine Serum Albumin (Sigma) as a protein standard.

### Statistical analysis

The enzyme activity and biomass data collected were conducted in triplicate and analysed separately for each fungal isolate using one-way analysis of variance (ANOVA). A significant difference between the means of each treatment was determined by least significant difference (LSD) test at the 0.05 level of significance. LSD analysis was conducted using a software by Assaad et al. (2014).

## Results

Fungal isolates were screened to determine their ability to produce endoglucanase. *Leohumicola* sp., EdRU083 and EdRU002 isolates were confirmed to have cellulolytic activity using the plating method.

### Effect of different pH on endoglucanase production

**Fig. 1:**
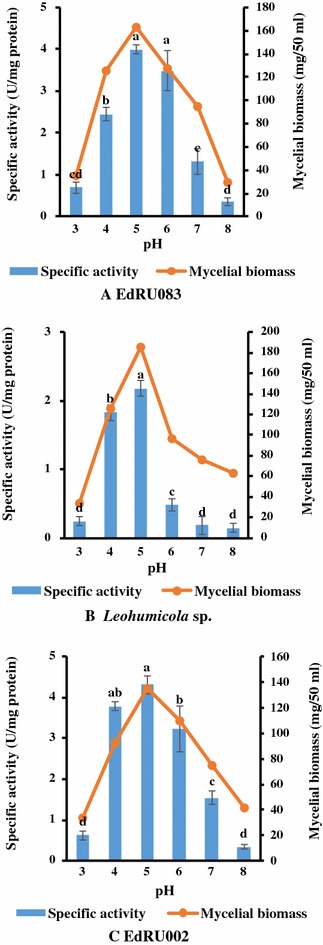
**A**–**C** Effect of different pH on endoglucanase production. All treatments were performed in triplicate (*p*  < 0.05). Means without a common *superscript letter* differ as analysed by one-way ANOVA and the LSD test while *error bar* indicated standard error of the mean

The effect of pH on enzyme and biomass production was conducted using HCl or NaOH at various pH (3.0, 4.0, 5.0, 6.0, 7.0 and 8.0) at 28 °C for 3 weeks and endoglucanase activity was assayed for using citrate–phosphate buffer (pH 5.0). Maximum enzyme activity and biomass yield were obtained between pH 4.0–6.0 (Fig. 1) except for *Leohumicola* sp. that showed a significant decrease in activity at pH 6.0. Further pH increase or decrease beyond this resulted in a significant decline of activity and biomass yield for all isolates.

### Effect of different carbon sources on endoglucanase production

**Fig. 2:**
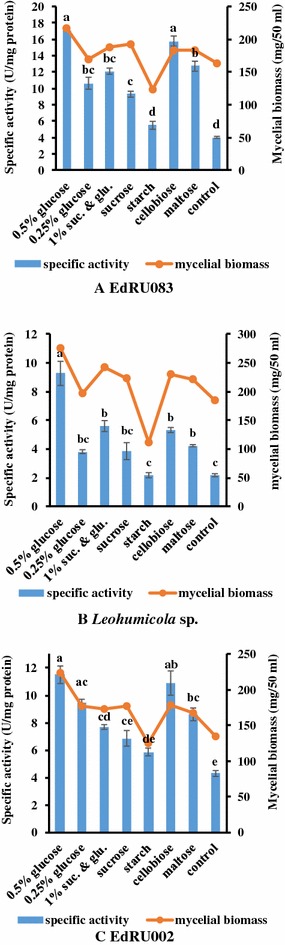
**A**–**C** Effect of different carbon sources on endoglucanase production. All treatments were performed in triplicate (p  < 0.05). Means without a common *superscript letter* differ as analysed by one-way ANOVA and the LSD test while *error bar* indicated standard error of the mean

Ericoid fungi were able to grow moderately on most of the tested carbon sources and increased endoglucanase production and yield of biomass were obtained. Glucose (0.5%) gave the highest enzyme activity followed by 1.0% cellobiose, 1.0% maltose, 1.0% sucrose/glucose, 0.25% glucose, 1.0% sucrose and 1.0% starch (Fig. 2). There was a significant difference in enzyme activity and mycelial biomass yield when the production medium was supplemented with 0.5% glucose and least when 1.0% starch was used as a supplement (Fig. 2). This indicates that the amount or type of carbon source in culture medium plays a role in determining the enzyme and biomass yield of all isolates.

### Effect of different nitrogen sources on endoglucanase production

**Fig. 3:**
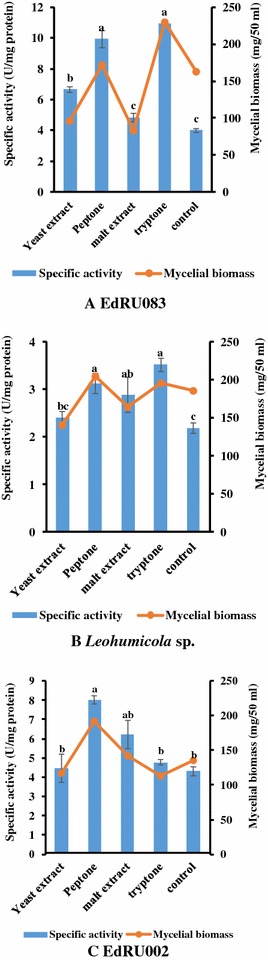
**A**–**C** Effect of different nitrogen sources on endoglucanase production. All treatments were performed in triplicate (p  < 0.05). Means without a common *superscript letter* differ as analysed by one-way ANOVA and the LSD test while *error bar* indicated standard error of the mean

Organic nitrogen has an inducing effect on the production of endoglucanase and biomass yield of all tested isolates. A significant increase in enzyme activity was noted when 1.0% tryptone was added to production medium for *Leohumicola* sp. and EdRU083 isolates while 0.1% peptone was significant for isolate EdRU002 (Fig. 3). Minimum activity was obtained when yeast extract or malt extract was used as a supplement.

### Effect of different metal ions on endoglucanase production

**Fig. 4:**
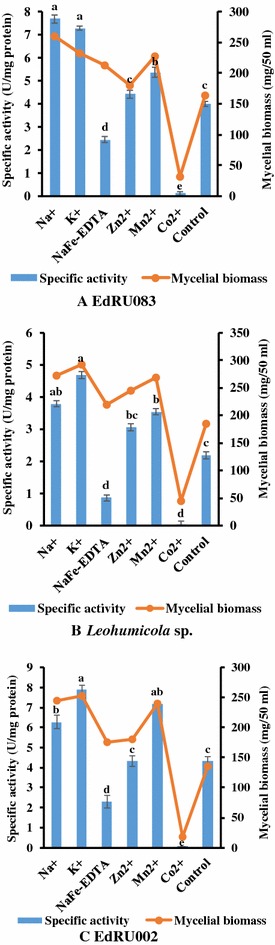
**A**–**C** Effect of different metal ions on endoglucanase production. All treatments were performed in triplicate (p  < 0.05). Means without a common *superscript letter* differ as analysed by one-way ANOVA and the LSD test while *error bar* indicated standard error of the mean

The effect of metal ions on the production of endoglucanase and biomass was noticeable when either 0.1% metal ion (Na^+^, K^+^, Mn^2+^, Zn^2+^, NaFe-EDTA or Co^2+^) was added to the liquid medium (Fig. 4). A significant enzyme activity was obtained when the medium was supplemented with potassium for all tested isolates followed by sodium while a significantly low activity was recorded when supplemented with cobalt (Fig. 4).

## Discussion

The pH of a growing medium is one of the most critical factors affecting fungal growth, enzyme production and transport of various components across the cell membrane (Juhasz et al. 2004). Thus, accounts for growth, reproduction and morphology of microorganisms in a production medium. High enzyme activities of 3.99, 2.18 and 4.31 (U/mg protein) were obtained for EdRU083, *Leohumicola* sp. and EdRU002 respectively at the pH of 5.0 with a corresponding mycelial biomass of 163, 185 and 135 mg/50 mL respectively (Fig. 1). This is similar to reports of Yazdi et al. (1990), Manavalan et al. (2015) and Saini et al. (2015) who recorded an optimal pH of 4.5 for endoglucanase activity (crude extract) for *Neurospora crassa*, pH 5.0 for *Penicillium oxalicum*, and pH 5.0 for purified enzyme from *Ganoderma lucidum*, respectively. Conversely, El-Hadi et al. (2013) also reported that a maximum CMCase production of 0.23 U/mL was achieved at pH 7.0 in a liquid medium and assay temperature of 37 °C for *Aspergillus hortai*. The effect of pH on endoglucanase production by these fungi (Fig. 1) aligned also with the observations made by Lynd et al. (2002) who reviewed studies on endoglucanase and filter paper activities exhibit and concluded that pH between pH of 4.0 and 7.0 was optimal for most microbes.

The three ericoid fungi selected for this study produced highest enzyme activities and mycelial biomass when the production medium was supplemented with 0.5% glucose (Fig. 2). Conversely, least endoglucanase activity and mycelial yield were obtained when there was no other carbon supplementation (control) except 0.5% CMC was used. This agrees with the work of Cao and Crawford (1993), where four strains of an ectomycorrhizal fungus, *Pisolithus tinctorius* was investigated for carbon nutrition, and for the production of hydrolytic and cellulolytic enzymes. Glucose, maltose, and cellobiose supported the rapid mycelial growth of all the four strains studied. This indicates that the enzyme expressions of these fungi were heavily influenced by the substrate they colonised. Cao and Crawford (1993) also reported the presence of an inducible cellulolytic enzyme complex in some ectomycorrhizal fungi, grown in vitro with similar medium composition. Recently, Bizabani and Dames (2016) reported high biomass productions when some ericaceous fungi (*Meliniomyces* sp., *Acremonium implicatum*, *Leohumicola* sp., *Cryptosporiopsis erica*, *Oidiodendron maius* and an unidentified Helotiales fungus) were grown on glucose and cellobiose. Thus, we affirmed that glucose and cellobiose (Fig. 2) were efficient at improving enzyme activity when compared with other fermentable sugars, and their incorporation into endoglucanase production media should be encouraged for improved ERM and DSE mycelial production.

Organic nitrogen sources have been reported to have an inducing effect on the production of cellulases (Jonathan and Adeoyo 2011a, b). This effect was discovered to be highest when the production medium was supplemented with peptone and tryptone (Fig. 3). These observations were similar to the findings of Jonathan and Adeoyo (2011a, b) who reported highest cellulase activity and mycelial biomass when the production medium was supplemented with peptone, recording values of 0.74 U/mL and 116 mg/30 mL respectively for *Coriolus versicolor*. The report of El-Hadi et al. (2013) also confirmed the role of organic nitrogen (e.g. peptone) at increasing enzyme activity during optimization of cultural and nutritional conditions for carboxymethylcellulase production by *Aspergillus hortai*, stating that organic nitrogen sources are preferable for improving endoglucanase activity. Similarly, Gautam et al. (2011) reported that either peptone or yeast extract (1.0%, w/v) was found to be the best nitrogen source for the production of cellulase by *Aspergillus niger* and *Trichoderma* sp.

Endoglucanase activity for most isolates used for this study was increased in the presence of metal ions like Na^+^ K^+^ Mn^2+^, followed by Zn^2+^, NaFe-EDTA and least when Co^2+^ was used to supplement the liquid medium. A low activity and mycelial biomass obtained for both NaFe-EDTA and Co^2+^ may be due to their toxic effect on these fungi during assimilatory processes. The report of Baldrian and Gabriel (2002) showed that a white-rot fungus (*Pleurotus ostreatus*) required trace amounts of essential heavy metals (e.g. Cd, Mn or Zn) for enzyme production and was corroborated by the findings of Falih (1998) and Baldrian (2003) who reported that metals in higher concentrations are potent inhibitors of enzymatic reactions (e.g. cellulase activity) in white-rot fungi. Based on current results (Fig. 4), cobalt ion and ferric EDTA concentration of 0.1% or more is inhibitory to the production of endoglucanase and biomass yield in both ERM and DSE fungi. Tolerance to metals has been reported to be species and metal dependent as shown by Berthelot et al. (2016) who investigated minimum inhibitory concentrations of As, Cd, Mn and Zn on fungal growth of several DSE strains. Two unidentified Helotiales isolates were shown to exhibit a high tolerance to Cd, Cu, Pb and Zn (Lacercat-Didier et al. 2016). These interspecific variations in fungal growth may similarly affect endoglucanase activity.

In conclusion, the potential of using ericoid fungi in producing improved mycelial biomass and endoglucanase activity in vitro under various pH and nutritional conditions were investigated in order to maximize enzyme production. This investigation provides useful information that could be helpful in improving the mycelial biomass yield of some selected starter culture for better mycorrhizal fungus inoculum production, while the endoglucanase activity can contribute to the improvement of cellulose bioconversion. Genome studies of ericoid mycorrhizal fungi such as *Oidiodendron maius* (Kohler et al. 2015) and *Cairneyella variabilis* (Midgley et al. 2016) have indicated an array of genes required for degradative catabolism. Therefore, bio-enzymes obtained from these fungi represent a potential additional source of endoglucanase for industrial applications. Based on the information currently available, this is the first report which shows the potential of ericoid fungi for commercial endoglucanase production. Finally, a MMN medium composition of pH 5.0 with 0.5% glucose, 1.0% peptone or tryptone and either (0.1%) sodium, potassium or manganese sulphate is adequate for the production of endoglucanase from ERM and DSE fungi.

## Abbreviations

ERM: ericoid mcyorrhizal
DSE: dark septate endophytes
CMC: carboxylmethylcellulose
MEA: malt extract agar
MMN: Modified Melin Norkrans
DNS: dinitrosalicyclic acid
ANOVA: analysis of variance
LSD: least significant difference
